# Polychlorinated Biphenyls and Their Hydroxylated Metabolites (OH-PCBs) in Pregnant Women from Eastern Slovakia

**DOI:** 10.1289/ehp.8913

**Published:** 2006-08-22

**Authors:** June-Soo Park, Linda Linderholm, M. Judith Charles, Maria Athanasiadou, Jan Petrik, Anton Kocan, Beata Drobna, Tomas Trnovec, Åke Bergman, Irva Hertz-Picciotto

**Affiliations:** 1 Department of Public Health Sciences, University of California-Davis, Davis, California, USA; 2 Department of Toxic Substances Control, California Environmental Protection Agency, Berkeley, California, USA; 3 Department of Environmental Chemistry, Stockholm University, Stockholm, Sweden; 4 Department of Environmental Toxicology, University of California-Davis, Davis, California, USA; 5 Department of Toxic Organic Pollutants, Slovak Medical University, Bratislava, Slovak Republic

**Keywords:** hydroxylated PCB metabolites, maternal blood serum, OH-PCBs, PCBs, PCP, penta-chlorophenol, polychlorinated biphenyls, Slovakia

## Abstract

**Objective:**

Our aim in the present study was to characterize and quantify the levels of polychlorinated biphenyls (PCBs) and specific polychlorobiphenylol (OH-PCB) metabolites in maternal sera from women delivering in eastern Slovakia.

**Design:**

During 2002–2004, blood samples were collected from women delivering in two Slovak locations: Michalovce district, where PCBs were formerly manufactured, and Svidnik and Stropkov districts, about 70 km north.

**Participants:**

A total of 762 and 341 pregnant women were sampled from Michalovce and Svidnik/Stropkov, respectively, and OH-PCBs were measured in 131 and 31.

**Evaluation/Measurements:**

We analyzed PCBs using gas chromatography (GC)/electron capture detection. OH-PCBs and pentachlorophenol (PCP) were determined as methyl derivatives using GC-electron capture negative ionization/mass spectrometry. We characterized distributions in the full cohort using inverse sampling weights.

**Results:**

The concentrations of both PCBs and OH-PCB metabolites of Michalovce mothers were about two times higher than those of the Svidnik/Stropkov mothers (*p* < 0.001). The median weighted maternal serum levels of the sum of PCBs (∑PCBs) were 5.73 ng/g wet weight (Michalovce) and 2.82 ng/g wet weight (Svidnik/Stropkov). The median sum of OH-PCBs (∑OH-PCBs) was 0.55 ng/g wet weight in Michalovce mothers and 0.32 ng/g wet weight in Svidnik/Stropkov mothers. 4-OH-2,2′ ,3,4′ ,5,5′ ,6-Heptachlorobiphenyl (4-OH-CB187) was a primary metabolite, followed by 4-OH-2,2′ ,3,4′ ,5,5′ -hexachlorobiphenyl (4-OH-CB146). Only four PCB congeners—CBs 153, 138, 180, and 170—had higher concentrations than 4-OH-CB187 and 4-OH-CB146 (*p* < 0.001). The median ratio of the ∑OH-PCBs to the ∑PCBs was 0.10.

**Conclusions:**

Mothers residing in eastern Slovakia are still highly exposed to PCBs, and their body burdens of these pollutants and OH-PCB metabolites may pose a risk for adverse effects on health for themselves and their children.

The concentrations of polychlorinated biphenyls (PCBs) have, in general, decreased in both humans and wildlife (Bignert et al. 1998; Norén and Meironyte 2000; Norstrom et al. 1988; Schade and Heinzow 1998). However, their high chemical stability, persistence, pronounced lipophilicity, and tendency to bioaccumulate, as well as their extensive technical production and use from 1929 to the 1970s, still make PCBs one of the world’s most widespread and problematic classes of environmental contaminants (Erickson 2001; Safe 1993).

PCB concentrations found in humans vary greatly, but commonly they are in the hundreds of nanograms per gram of lipid if presented as the sum of PCBs (∑PCBs) (James et al. 2002; Sjödin et al. 2000). Higher PCB concentrations have been observed for populations exposed to PCBs via certain food sources, such as sea mammals and/or fish (Asplund et al. 1994; Ayotte et al. 1997; Fängström et al. 2002; He et al. 2001; Hsu et al. 1985; Sandau et al. 2000; Sjödin et al. 2000), or other routes of exposure. Accidental intake of PCBs may cause elevated concentrations of this class of compounds, as was the case in Belgium in 1999 when animal feeds were accidentally contaminated by PCBs (Bernard and Fierens 2002; Bernard et al. 1999). Toxicologic effects of PCBs observed in animal studies include carcinogenicity, reproductive impairment, neurodevelopmental anomalies, and immunologic deficiency (Brouwer et al. 1998; Faroon et al. 2001a, 2001b; Seegal 1996). However, not all of these outcomes have been shown in humans. Moreover, it is not clear whether effects observed in humans were caused by the PCBs themselves or by related compounds. Jacobson and Jacobson (1996, 2004) reported adverse effects of prenatal PCB exposure (> 1.25 μg/g lipid) on neurodevelopment and intellectual behavior in infants and young children from Michigan, and similar results have been reported in cohorts living elsewhere (Patandin et al. 1999; Stewart et al. 2004; Winneke et al. 2002).

Over the last decade attention has been drawn to OH-PCBs that are metabolites of PCBs and their potential role in early development (Brouwer et al. 1998; Letcher et al. 2000; Meerts et al. 2004a, 2004b). OH-PCB metabolites are predominantly formed from PCB congeners via cytochrome P450 (CYP450)-mediated oxidation (Letcher et al. 2000). The hydroxyl group on a biphenyl is in general inserted via arene oxide formation, possibly followed by 1,2 shift (NIH shift) (Guroff et al. 1967; Jerina and Daly 1974). Alternatively OH-PCB metabolites may be formed via direct insertion of the hydroxyl group (Koga et al. 1995). The metabolism of PCB congeners results in the formation of a very large number of OH-PCB congeners (Bergman et al. 1994; Letcher et al. 2000; Sjödin et al. 1998). OH-PCB metabolites with a *para*- or a *meta*-substituted hydroxyl group with adjacent chlorine atoms in the *ortho* and *meta* positions have a high affinity for transthyretin (TTR), a thyroxine (T_4_) transport protein (Lans et al. 1993). A set of structurally known OH-PCB metabolites are strong T_4_ competitors for TTR (Brouwer et al. 1990, 1998; Lans et al. 1993, 1994; Purkey et al. 2004). Due to the ability of TTR to pass the placental and blood–brain barriers, OH-PCBs can be preferentially transferred to the fetal compartment. In rodents, where TTR is the only T_4_ binding protein (Brouwer et al. 1998; Darnerud et al. 1996; Meerts et al. 2002; Morse et al. 1996; Sinjari and Darnerud 1998), OH-PCBs induce hypothyroidism in brain tissue. TTR is one of three T_4_ carriers in human blood. In addition, OH-PCBs may cause fetal hypothyroidism through disruption of T_4_ homeostasis by deiodination (Brouwer et al. 1998) and sulfation (Schuur et al. 1998a, 1998b). OH-PCBs have recently been shown to be more efficiently transferred to the fetal compartment than their parent compounds (Meironyté Guvenius et al. 2003; Soechitram et al. 2004).

We conducted this investigation as part of a birth cohort study of PCBs and early childhood development. Details on the study design and background have been presented elsewhere (Hertz-Picciotto et al. 2003). The Chemko, Inc., chemical plant located in the Michalovce district of the Slovak Republic produced PCBs under the name of “Delor” from 1959 to 1984. A large quantity of PCB waste generated during the PCB manufacture was released into the surrounding area by improper disposal, resulting in high environmental PCB levels in various matrices in the Michalovce district, for example, in air, soil, sediment, water, fish, and wildlife (Kocan et al. 2001). Concentrations of PCBs in human blood were far higher than in other areas of Slovakia (Hovander et al. 2006; Kocan et al. 1994; Pavuk et al. 2004).

The objective of the present study was to assess OH-PCB and PCB congener concentrations and patterns in mothers delivering in two districts of eastern Slovakia. Ultimately, we hope to improve understanding of the relationship between OH-PCBs and immune and neurobehavioral development. We characterized and quantified the levels of specific OH-PCB metabolites in maternal sera collected at delivery. Also, we analyzed pentachlorophenol (PCP) because it is known to be a major blood contaminant (Hovander et al. 2002; Meironyté Guvenius et al. 2003; Sandau et al. 2000).

## Materials and Methods

### Cohorts and samples

We collected blood samples from mothers delivering in the Michalovce district (population, 110,000), home to a chemical manufacturing facility (Chemko Strazske) that produced PCBs from 1959 to 1984, and from the Svidnik and Stropkov districts (population, 55,000), which have lower PCB exposures but similar population characteristics (Demographic Research Center, Bratislava). During the period 2002–2004, mothers delivering in these areas were enrolled in a birth cohort study. Hospital staff trained in the study protocols explained the study and obtained consent before collection of data, and collected blood samples from mothers at time of delivery. The study participants gave written informed consent. A total of 762 and 341 women were sampled from Michalovce and Svidnik/Stropkov, respectively. This study complied with all applicable U.S. and international requirements with regard to research on human subjects and was approved by the respective institutional review boards at the University of California-Davis and the Slovak Medical University (formerly The Institute for Preventive and Clinical Medicine).

Whole maternal blood (20 mL) was collected from subjects just before the delivery into three 9-mL vacutainer tubes (S-Monovette, Sarstedt, Germany) without anticoagulants. The blood samples were allowed to clot ≤ 2 hr at 5–10°C. After clotting, blood was centrifuged at 3,000 rpm for 15 min. Isolated serum was stored frozen at –18°C in pre-cleaned glass tubes with polytetrafluoroethylene liner screw caps. We transported all the samples by car to the Slovak Medical University (SMU) laboratory in Bratislava in thermo boxes to prevent thawing. We stored the samples in freezers at –18°C until analysis. Aliquots of 5 mL were shipped to the University of California-Davis and stored at –80°C until analysis of OH-PCB metabolites. We asked the mothers who participated in this study to complete questionnaires (e.g., age, diet, number of children, residence, lifestyle, ethnicity, education). Based on the maternal serum concentrations of PCBs measured for this study, we selected a stratified random sample of the specimens for the analysis of OH-PCB metabolites. We assigned probabilities of selection such that those at the low end of the PCB distribution had the lowest probability of selection, and those at the upper end had the highest probability.

### Chemicals and instruments

The organic solvents and adsorbents used in the analysis and the authentic reference standards used for the identification and quantification of the analytes are described in Table 1. We followed the numbering of OH-PCBs as proposed elsewhere (Hovander et al. 2002). OH-PCBs and PCP in the final extracts were determined as methyl derivatives by using an Agilent 6890 gas chromatograph (Agilent Technologies, Palo Alto, CA, USA) equipped with an Agilent 5973N mass spectrometer (Agilent Technologies). Individual methyl derivatives prepared after methylation of authentic reference standards were injected in the gas chromatograph (GC)/mass spectrometer operated in electron capture negative ionization (ECNI) full scan mode. The observed mass spectra and retention time were used for identification of the analytes. Helium was used as carrier gas. The mass spectrometer was operated in ECNI mode with electron energy of 130 eV. Methane (99.99% pure) was used as a reagent gas, and source pressure was 2.0 × 10–4 torr at the inlet. The GC was equipped with DB-5MS capillary column (30 m × 0.25 mm i.d., 0.25 μm film thickness; J&W Scientific Inc., Folsom, CA, USA). Injection (2 μL) was made in pulsed splitless mode with an injector temperature of 250°C. The initial GC temperature was set to 80°C and held for 2 min, followed by a 50°C/min increase to 200°C, 1°C/min to 230°C, and 30°C/min to 300°C, and held for 4 min. Postrun was set to 320°C for 1 min. The temperatures for both ion source and quadruple field were set to 150°C. Selective ion monitoring (SIM) was used to monitor two ions for each compound: one for quantification and the other for confirmation. For most OH-CB congeners and PCP, the molecular ions were monitored as the base peak. However, the most abundant fragment ions [(M+2-HCl)^–^] were monitored for *meta*-substituted congeners (e.g., 3′ -MeO-CB138 and 3-MeO-CB153) because their molecular ions were weak in intensity.

### Analysis and cleanup

We analyzed maternal blood sera specimens for 17 PCB congeners (CBs 28, 52, 101, 123/149, 118, 114, 153, 105, 138, 167, 156/171, 157, 180, 170, and 189) at the Slovak Medical University. The analytical procedures are described in detail elsewhere (Kocan et al. 1994; Pavuk et al. 2004). The total lipid content was measured enzymatically (Akins et al. 1989). We analyzed the selected samples (*n* = 166) for nine OH-PCB congeners and PCP at the University of California-Davis. Maternal sera specimens were weighed in precleaned glass tubes (25 mL) with polytetrafluoroethylene liner screw caps. The extraction method was adopted to separate phenolic compounds (e.g., OH-PCBs, PCP) from maternal serum (Hovander et al. 2000). The procedures of methyl derivatization and purification were optimized for this study. Briefly, maternal serum (5 g) was denaturized with 1 mL of 6 M hydrochloric acid and 6 mL 2-propanol and extracted using 6 mL hexane:methyl-*tert*-butyl ether (MTBE) (1:1; reextraction with 3 mL). After potassium chloride wash, the extracts were phase-separated by adding 2 mL potassium hydroxide solution. After the neutral fraction was extracted, the alkaline aqueous solutions (potassium hydroxide) were acidified with 2 M hydrochloric acid and extracted for phenolic compounds with 4 mL hexane:MTBE (9:1; reextraction with 3 mL). The phenolic extracts were further dried by passing them through anhydrous sodium sulfate–packed Pasteur pipette columns. The volumes of phenolic extracts and five calibration standards were reduced to < 50 μL. Five drops of methanol were added to all sample extracts to complete the derivatization and to give the extracts similar properties to calibration standards in methanol. Phenolic compounds in the sample extracts and calibration standards were methylated by adding 1 mL diazomethane. We synthesized diazomethane in hexane by using *N*-nitroso-*N*-methylurea (Sigma-Aldrich, St. Louis, MO, USA) as described by Sandau (2000). The glass tubes containing the mixtures were tightly capped, placed in a dark box, and left for 24 hr in a ventilation hood.

In this study, we compared two different cleanup methods to improve the removal of any coextracted biogenic residues. For the initial 60 samples, the derivatives were purified on a 22% sulfuric acid (H_2_SO_4_)/silica column (1.5 g) with 40 mL dichloromethane (DCM):hexane (1:1) as the mobile phase, using custom-made glass columns (23 cm × 0.6 cm i.d.) with a 50-mL reservoir. For the rest of the samples (*n* = 106), the extracts were first treated with concentrated H_2_SO_4_ (98%) and then further cleaned up with 1:2 H_2_SO_4_:silica gel (weight/weight; 0.5 g) column and 10 mL DCM:hexane (1:1) as mobile phase. For both methods, the silica gel was activated at 550°C. The volumes of eluates and calibration standards were reduced to 200 μL as a final volume, spiked with CB-209 (3.15 ng) as an injection standard, and transferred to brown GC vials with silanized inserts.

### Quantification and quality assurance/quality control

All glassware was washed, dried by acetone and hexane, and baked at 550°C for 8 hr. For the analysis of OH-PCB metabolites, each batch included 1 reagent blank (1% potassium chloride solution), 1–2 control samples, and 10 serum specimens. Five levels of calibration standards for the quantification were prepared in methanol and stored in brown ampules in a refrigerator. The calibration range was 0.1–50 pg/μL. For control samples, we obtained human adult serum samples (*n* = 3) from the Center for Blood Research (Sacramento, CA, USA); the samples were pooled and measured for PCP and OH-PCBs. Subsequently, PCP and OH-PCB standards were fortified to the pooled serum. We used the sum of measured and fortified compounds as a final concentration to determine the recoveries of analytes. After several recovery tests on the pooled serum for consistency, it was aliquoted in glass tubes, wrapped in aluminum foil, and stored at –20°C until analysis. For the quantification, we used five OH-PCB external calibration standards derivatized simultaneously with the serum sample extracts for accurate quantification. 4′ -OH-CB159 (4′ -OH-2,3,3′ ,4,5,5′ -hexachlorobiphenyl; 2.00 ng) was added to all samples before extraction as a recovery internal standard.

### Statistical analysis

Because we selected the subsample for OH-PCB assays based on strata defined by PCB concentration, the distribution of PCBs and OH-PCB concentrations were represented by their means and medians obtained using weights proportional to the inverse of the sampling fractions. However, we report the measured concentrations of PCP as randomly selected because PCP was not associated with PCBs. We report the concentration of phenolic compounds, OH-PCBs, and PCP on a wet weight (ww) basis because they are not accumulated in lipids but have high affinity to blood proteins (Bergman et al. 1994; Letcher et al. 2000). We conducted parametric tests for log-normally distributed PCB and OH-PCB data to determine the differences and relationships. After log transformation, we calculated *R*^2^ values from a weighted Pearson correlation analysis to determine the relationships between individual PCBs and their presumed metabolites in maternal serum. After log transformation, we conducted Student *t*-tests to determine differences in the concentrations between Michalovce and Svidnik/Stropkov mothers, and between PCBs and their metabolites. We used the nonparametric Mann-Whitney test for PCP data, which were not normally distributed.

## Results

**Figure 1:**
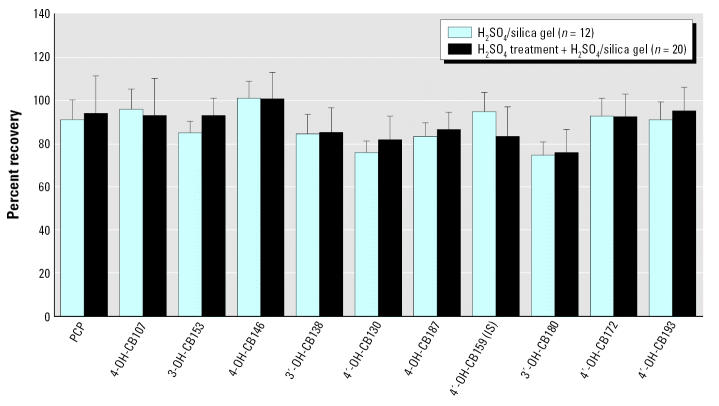
Percent recovery of OH-PCBs from spiked control serum samples applying two different cleanup procedures. IS, internal standard.

We report results for nine OH-PCB congeners, their sum, and PCP for the 166 maternal serum specimens. The recoveries from control samples in a total of 18 batches ranged from 75 ± 9% [4′ -OH-CB130 (4′ -OH-2,2′ ,3,3′ ,4,5′ -hexachlorobiphenyl)] to 101 ± 11% [4-OH-CB146 (4-OH-2,2′ ,3,4′ ,5,5′ -hexachlorobiphenyl)]. The differences between the two cleanup methods with regard to the recovery of analytes from control samples are shown in Figure 1. Most recoveries of the internal standard (4′ -OH-CB159) fell between 60 and 110%, with an average of 84 ± 16%. Twenty samples had lower recoveries (40–60%). Of 166 samples, 4 were omitted from the statistical summary: 3 because the recoveries of internal standards were < 30%, and 1 because the PCB concentrations in this Svidnik/Stropkov mother were far outside the range for other mothers in the districts, raising concern about contamination. We corrected all other serum concentrations of OH-PCBs using the recoveries of internal standards and subtracting the quantity measured in each reagent blank analyzed in the same batch.

Table 2 presents demographic and lifestyle characteristics, including age, parity, body mass index, education, marital status, and smoking during pregnancy for the 131 subjects from Michalovce district and the 31 from Svidnik/Stropkov districts. Compared with the total population in this study, the Michalovce subsample has more first-time mothers (data not shown). The concentrations of lipids, various individual and summed PCBs and OH-PCBs, and PCP are presented in Tables 3 and 4. Lipid content in maternal serum was similar in samples from Michalovce compared with those from Svidnik/Stropkov, with medians of 1.02% and 1.01%, respectively. The mean concentrations of both PCBs and OH-PCB metabolites in sera from Michalovce mothers were about twice as high as in sera from Svidnik/Stropkov mothers (*p* < 0.001). The ratios tended to be even higher at the 95th percentiles. Median maternal serum levels of the ∑PCBs were 5.73 ng/g ww in Michalovce and 2.82 ng/g ww in Svidnik/Stropkov (Table 3). The medians for the sum of nine measured OH-PCB metabolites were 0.55 ng/g ww in Michalovce mothers and 0.32 ng/g ww in Svidnik/Stropkov mothers (Table 4). 4-OH-CB187 (4-OH-2,2′ ,3,4′ ,5,5′ ,6-heptachlorobiphenyl) was the predominant congener in almost all samples, followed by 4-OH-CB146. 4-OH-CB187 comprised 36 ± 6% of the ∑OH-PCBs we measured, and combined with 4-OH-CB146, accounted for 57 ± 8%. These two OH-PCBs, combined with four other metabolites [3-OH-CB153 (3-OH-2,2′ ,4,4′ ,5,5-hexachlorobiphenyl), 3′ -OH-CB138 (3′ -OH-2, 2 ′, 3,4, 4 ′, 5 ′ - hexachlorobiphenyl), 4′ -OH-CB172 (4′ -OH-2,2′ ,3,3′ ,4,5,5′ -heptachlorobiphenyl), and 4-OH-CB107 (4-OH-2,3,3′ ,4′ ,5-pentachlorobiphenyl)], constituted 93 ± 3% of the ∑OH-PCBs, whereas 4′ -OH-CB130, 3′ -OH-CB180 (3′ -OH-2,2′ ,3,4,4′ ,5,5′ -heptachlorobiphenyl), and 4-OH-CB193 (4-OH-2,3,3′ ,4′ ,5,5′ ,6-heptachlorobiphenyl) were detected only at trace levels in most samples. The median PCP concentrations were 0.66 ng/g ww for the women from Michalovce and 0.54 ng/g ww for the Svidnik/Stropkov subjects.

**Figure 2:**
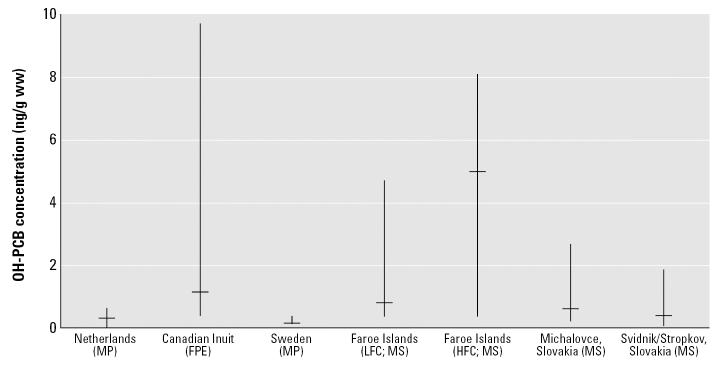
Medians and ranges of maternal blood concentrations of major OH-PCB metabolites from five other areas for comparison to present data. Abbreviations: FPE, female plasma equivalent; HFC, high fish consumption; LFC, low fish consumption; MP, maternal plasma; MS, maternal serum. The samples were collected for the Netherlands in 1998–2000 (Soechitram et al. 2004), Canadian Inuit in 1992 (Sandau et al. 2000), Sweden in 2000–2001 (Meironyté Guvenius et al. 2003), Faroe Islands in 1994–1995 (Fängström et al. 2002), and Michalovce and Svidnik/Stropkov in 2002–2004 (present study). The distribution for the Slovak region represents the 1,103 mothers through weighting based on the sample design.

The sum of six major OH-PCB metabolites (see Table 4 footnote) from five different geographic locations are summarized as median or equivalent value (geometric mean) and range (Figure 2): 0.52 and 0.30 ng/g ww for mothers residing in Michalovce and Svidnik/Stropkov, respectively (this study); 0.32 for mothers from the northern Netherlands (Soechitram et al. 2004); 1.11 for female Inuit in northern Canada (Sandau et al. 2000); 0.12 for Swedish mothers (Meironyté Guvenius et al. 2003); and 0.75 and 5.00 for low- and high-exposed Faroe Island mothers, respectively (Fängström et al. 2002). Concentrations of OH-PCB metabolites from Michalovce mothers were comparable to those from Faroe Island mothers with low fish consumption. In Figure 2, data on 3′ -OH-CB138 was not included for northern Canadian female Inuit because it was detected only at trace levels (~ 3%). No data from Faroe Island mothers is available for 4′ -OH-CB172 because Fängström et al. (2002) did not measure it. Although data from Swedish mothers provided the sum of 12 OH-PCB metabolites (Meironyté Guvenius et al. 2003), the concentrations were lowest among the published studies. Faroe Island mothers in 1994–1995 (Fängström et al. 2002), especially frequent fish eaters, showed the highest median concentration of OH-PCB metabolites—4.5 times higher than northern Canadian female Inuits in 1992 and 9.0 times higher than Michalovce mothers during 2002–2004. However, Canadian female Inuit showed the widest concentration range.

**Figure 3:**
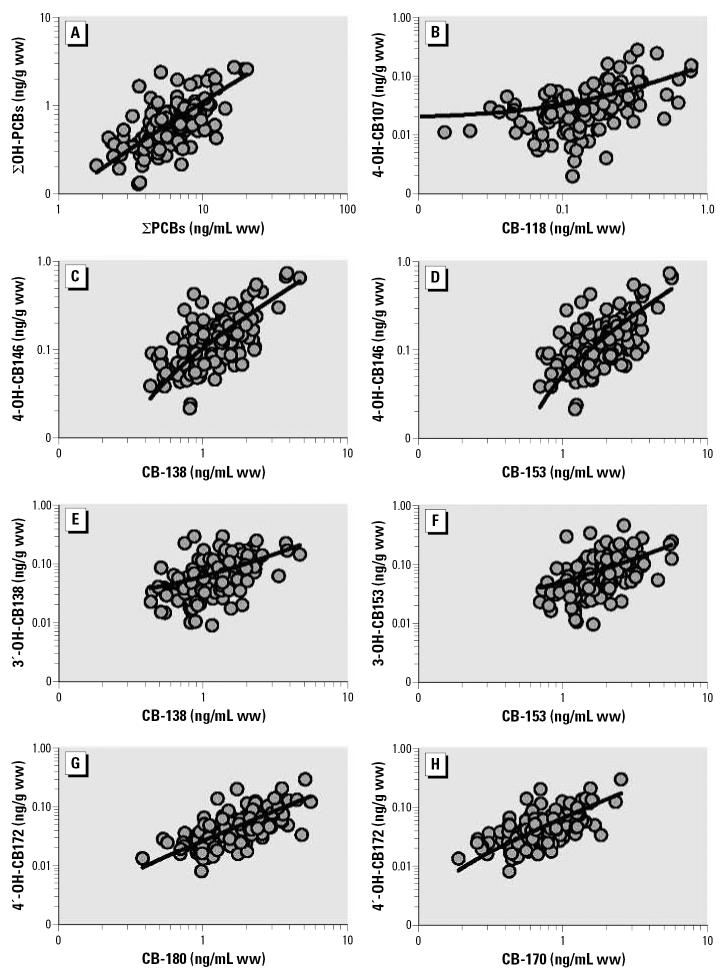
Best fitting linear regressions indicating statistical relationships between ∑PCBs and ∑OH-PCBs (*A; R*^2^ = 0.47) and between specific PCB precursors and OH-PCB metabolites in maternal sera from Slovakia: (*B*) CB-118 and 4-OH-CB107 (*R*^2^ = 0.24); (*C*) CB-138 and 4-OH-CB146 (*R*^2^ = 0.53); (*D*) CB-153 and 4-OH-CB146 (*R*^2^ = 0.52); (*E*) CB-138 and 3′ -OH-CB138 (*R*^2^ = 0.22); (*F*) CB-153 and 3-OH-CB153 (*R*^2^ = 0.22); (*G*) CB-180 and 4′ -OH-CB172 (*R*^2^ = 0.39); and (*H*) CB-170 and 4′ -OH-CB172 (*R*^2^ = 0.43).

The sum of OH-PCB metabolites correlated with the ∑PCBs (*R*^2^ = 0.47, *p* < 0.001) (Figure 3). The ratio of the ∑OH-PCBs to the ∑PCBs ranged from 0.03 to 0.47, with a median of 0.10. We also examined OH-PCB metabolites in relation to potential or known PCB precursors described by others (Letcher et al. 2000; Sjödin et al. 1998). For instance, 4-OH-CB107 was transformed from CB-105 and CB-118; both CB-138 and CB-153 were precursors for 4-OH-CB146; CB-170 and 180 were suggested as possible substrates for 4′ -OH-CB172; and 3-OH-CB153 and 3′ -OH-CB138 could be derived directly from CB-153 and CB-138, respectively. We used a correlation analysis to evaluate these biotrans-formation pathways of PCB congeners. These are shown in Figure 3; *R*^2^ values ranged from 0.22 to 0.53.

The median concentration of the ∑PCBs was an order of magnitude higher than that of OH-PCBs (*p* < 0.001). However, the most dominant OH-PCB metabolite, 4-OH-CB187, showed significantly higher concentration than CBs 28, 52, 101, 123/149, 114, 105, 167, 157, and 189 (*p* < 0.001 from all individual tests). It was slightly higher than CBs 118 and 156/171 but the difference was still significant (*p* < 0.001 from all individual tests). Only CBs 153, 138, 180, and 170 were significantly higher than 4-OH-CB187 (*p* < 0.001 from all individual tests).

**Figure 4:**
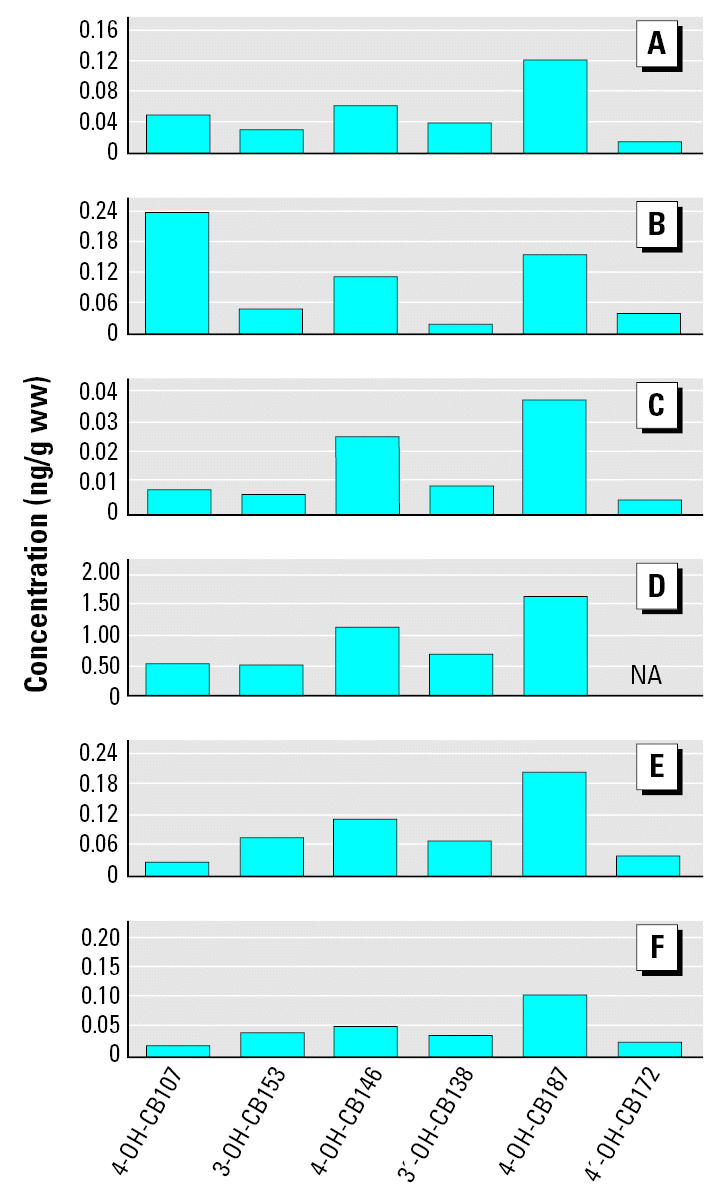
Distribution of major OH-PCB metabolites found in human blood for six regions in Europe and North America. (*A*) Netherlands (Soechitram et al. 2004), (*B*) Canadian Inuit (Sandau et al. 2000), (*C*) Sweden (Meironyté Guvenius et al. 2003), (*D*) Faroe Islands (Fängström et al. 2002), (*E*) Michalovce (present study), and (*F*) Svidnik/Stropkov (present study). NA, not available.

The distributions of six individual OH-PCB congeners in studies from various locations are shown in Figure 4. The relative abundances of these congeners are similar, with the exception of 4-OH-CB107, which contributes a larger proportion among the Inuits.

## Discussion

We found that 4-OH-CB107, 4-OH-CB187, 4-OH-CB146, 3-OH-CB153, 3′ -OH-CB138, and 4′ -OH-CB172 were the major hydroxylated PCB metabolites in most maternal serum specimens collected from the two regions (Michalovce and Svidnik/Stropkov districts). Many other OH-PCB congeners have also been identified in human blood (Hovander et al. 2002; Sandau et al. 2000). With only slight variation, the rank order of abundance of individual OH-PCB metabolites we observed in the present study was 4-OH-CB187 > 4-OH-CB146 > 3-OH-CB153 > 3′ -OH-CB138 > 4′ -OH-CB172 > 4-OH-CB107. If we can assume similar patterns of metabolic pathways, the identical pattern of OH-PCB congeners observed in the two regions might indicate atmospheric transport, similar upstream sources due to use of PCB-containing products in the past, or consumption of food contaminated over a wider area than Michalovce. The distribution of individual OH-PCBs varied geographically (Figure 4). We found 4-OH-CB187 to be a primary OH-PCB metabolite in the maternal blood, consistent with results from other studies (Bergman et al. 1994; Fängström et al. 2002; Klasson-Wehler et al. 1998; Meironyté Guvenius et al. 2003; Soechitram et al. 2004), whereas Sandau et al. (2000) observed 4-OH-CB107 to be a primary congener. Sjödin et al. (2000) also determined that 4-OH-CB107 was a primary congener in Baltic Sea fishermen. 4-OH-CB146 has consistently been found to be the second most abundant metabolite in all locations.

The correlation between PCBs and OH-PCB metabolites (*R*^2^ = 0.47, *p* < 0.001) indicated that the blood levels of OH-PCB metabolites were dependent on PCB levels. The ratio of the ∑OH-PCBs to the ∑PCBs was similar to that observed in other studies (Letcher et al. 2000; Meironyté Guvenius et al. 2003; Sandau et al. 2000; Sjödin et al. 2000; Soechitram et al. 2004). In other studies, lower ratios were sometimes related to higher levels of PCBs, which has been hypothesized to be due to faster elimination of OH-PCBs as a result of the enhanced induction of phase II enzymes and conjugation reactions (Letcher et al. 2000). In the present study we found no relationship between PCB levels and magnitude of the ratios. Several OH-PCBs can be formed from each of the persistent PCB congeners (e.g., CBs 118, 138, 153, 187) via a 1,2 shift of a chlorine atom in the hydroxylation stage or via a direct oxygen (hydroxyl group) insertion. Therefore, it is impossible to predict the relative contribution of PCB congeners to each specific OH-PCB metabolite. An important factor for the differences we observed must be the natural biological variability. The correlation analysis in the present study supports a 1,2 shift of a chlorine atom as a more important mechanism than direct insertion of oxygen in the formation of OH-PCBs. For example, pairs of 1,2 shift transformation (e.g., 4-OH-CB146/CB-138 in Figure 3C, 4-OH-CB146/CB-153 in Figure 3D) showed stronger correlation (*R*^2^ = 0.52–0.53) than those dominated by direct oxygen insertion (e.g., 3′ -OH-CB138/CB-138 in Figure 3E, 3-OH-CB153/CB-153 in Figure 3F) (*R*^2^ = 0.22). The CB-118 to 4-OH-CB107 relationship (Figure 3B) was weaker (*R*^2^ = 0.24) than other associations related to a 1,2 shift transformation. This may be due to its formation not only from CB-118 but also from CB-105. The relationship we observed between CB-118 and 4-OH-CB107 is slightly weaker than that reported in other studies (Sandau et al. 2000).

OH-PCB metabolites in Michalovce were comparable to those in Faroe Island mothers with low fish consumption (Figure 2). The Faroe Island mothers with high fish consumption showed much higher median concentration of OH-PCB metabolites than the subjects of any other areas, including Michalovce mothers and Canadian Inuit. The wide range among the Inuits is probably because of a wider age range (18–72 years) than in the other studies, as it was not limited to pregnant women. Generally, body burdens of PCBs are positively correlated with age because of accumulation and slow excretion, and accordingly, OH-PCBs should correlate as well. Unlike in the Slovak mothers, the high concentrations of OH-PCBs found in Canadian Inuit and Faroe Islanders (Figure 2) are related to ongoing fish and sea mammal consumption. Total PCBs in blubber of ringed seal and beluga whale (common in the Inuit diet) were 0.96–5.60 and 0.31–1.50 μg/g lipid, respectively (Sandau et al. 2000); the concentration of total PCBs in pilot whale blubber (Faroe Island diet) was 10–40 μg/g lipid (Fängström et al. 2002).

PCP was present in higher concentrations than any of the OH-PCB metabolites. This is in accordance with other studies (Meironyté Guvenius et al. 2003; Sandau et al. 2000). PCP has been extensively used as a fungicide for wood preservation, which may be the reason for its occurrence in our Slovakian subjects (Halsall et al. 1998; Sandala et al. 2004; Wild et al. 1992). PCP interferes with thyroid hormones in blood (Beard et al. 1999; Ishihara et al. 2003; van den Berg et al. 1991). However, in contrast to OH-PCB metabolites, the concentrations of PCP in the present study were not significantly different between the districts (Mann-Whitney test, *p* = 0.10); also, PCP showed no correlation with OH-PCB metabolites, supporting different sources of the HPCs. The median and geometric mean concentrations of PCP (0.63 and 0.68 ng/g ww, respectively) from eastern Slovak mothers were substantially lower than in Swedish mothers (median, 2.83; Meironyté Guvenius et al. 2003), and female Canadian Inuit (geometric mean, 1.59; Sandau et al. 2000).

The higher concentrations of both PCBs and OH-PCB metabolites in Michalovce compared with Svidnik/Stropkov is consistent with a cross-sectional study conducted just before ours. During 2001–2002, Hovander et al. (2006) analyzed six PCB congeners (CB-101, 105, 118,-138, 153, and 187) and three OH-PCB metabolites (4-OH-CB107, 4-OH-CB146, and 4-OH-CB187) in 122 plasma samples from Michalovce and 175 plasma samples from Svidnik/Stropkov from the general human population. They found median CB-153 concentrations of 0.57 μg/g lipid (Michalovce) and 0.24 μg/g lipid (Svidnik/Stropkov) compared with the values of 0.16 μg/g lipid (Michalovce) and 0.10 μg/g lipid (Svidnik/Stropkov) in our samples. Hovander et al. (2006) reported lipid-adjusted median 4-OH-CB187 concentrations of 0.13 μg/g (Michalovce) and 0.058 μg/g lipid (Svidnik/Stropkov), whereas we measured 0.02 and 0.01 μg/g lipid, respectively. When the two studies were compared for the concentrations of specific congeners of PCBs and OH-PCB metabolites, the study by Hovander et al. (2006) showed higher concentrations. However, the populations were not directly comparable, because the report of Hovander et al. was based on a sample of men and women 20–59 years of age, whereas our samples included a narrower, younger age range in women only. PCBs were strongly related to age (Kocan et al. 2004; Sandau et al. 2000) and additionally, blood lipid levels increase during the last half of pregnancy (Herrera et al. 2004).

In the present study we demonstrate that the population in the eastern Slovakia area is still exposed to high levels of environmental PCBs two decades after the production of PCBs stopped, including mothers who were born after cessation. We also found that this population carries significant concentrations of OH-PCB metabolites. Although maternal serum concentrations of OH-PCB metabolites in Svidnik/Stropkov were lower than in Michalovce, they were not trivial.

## Correction

Because of errors in calculations, summary statistics for concentrations of PCBs and OH-PCBs were incorrect in the manuscript originally published online. These errors were in the abstract, Tables 3 and 4, the text in the “Results” referring to these tables, and Figures 2 and 4. These errors have been corrected here.

## Figures and Tables

**Table 1:** Chemicals and authentic reference standards.

Chemical	Provider
Chemical (grade)	
Hexane (trace analysis)	Burdick & Jackson^a^
Dichloromethane (trace analysis)	Burdick & Jackson
Methyl-*tert* butyl ether (HPLC)	Fisher Scientific^b^
Methanol (purge and trap)	Fisher Scientific
Water (HPLC)	Burdick & Jackson
2-Propanol (99.9%, pesticide)	Sigma-Aldrich^c^
Hydrochloric acid	Fisher Scientific
H_2_SO_4_ (98%)	Fisher Scientific
Potassuim hydroxide	Fisher Scientific
Potassuim chloride	Fisher Scientific
Sodium hydroxide	Fisher Scientific
Ethyl alcohol (99.9%, 200 proof)	Gold Shield^d^
*N*-Nitroso-*N*-methylurea	Sigma-Aldrich
Silica (200–400 mesh)	Sigma-Aldrich
Standard (purity), chemical name^e^	
4-OH-CB107 (> 98%), 4-OH-2,3,3′ ,4′ ,5-pentachlorobiphenyl	Wellington Laboratory^f^
3-OH-CB153, 3-OH-2,2′ ,4,4′ ,5,5-hexachlorobiphenyl	Stockholm University^g^
4-OH-CB146 (> 98%), 4-OH-2,2′ ,3,4′ ,5,5′ -hexachlorobiphenyl	Wellington Laboratory
3′ -OH-CB138 (> 98%), 3′ -OH-2,2′ ,3,4,4′ ,5′ -hexachlorobiphenyl	Wellington Laboratory
4′ -OH-CB130 (> 98%), 4′ -OH-2,2′ ,3,3′ ,4,5′ -hexachlorobiphenyl	Wellington Laboratory
4-OH-CB187 (> 98%), 4-OH-2,2′ ,3,4′ ,5,5′ ,6-heptachlorobiphenyl	Wellington Laboratory
4′ -OH-CB159 (> 98%; IS), 4′ -OH-2,3,3′ ,4,5,5′ -hexachlorobiphenyl	AccuStandard^h^
3′ -OH-CB180 (> 98%), 3′ -OH-2,2′ ,3,4,4′ ,5,5′ -heptachlorobiphenyl	Wellington Laboratory
4′ -OH-CB172 (> 98%), 4′ -OH-2,2′ ,3,3′ ,4,5,5′ -heptachlorobiphenyl	Wellington Laboratory
4-OH-CB193, 4-OH-2,3,3′ ,4′ ,5,5′ ,6-heptachlorobiphenyl	Stockholm University
PCP (> 95%)	Ultra Scientific^i^

IS, internal standard.

a Muskegon, MI, USA.

b Pittsburgh, PA, USA.

c St. Louis, MO, USA.

d Hayward, CA, USA.

e The numbering of OH-PCBs follows that used by Hovander et al. (2002).

f Guelph, Ontario, Canada.

g Stockholm, Sweden.

h New Haven, CT, USA.

i North Kingstown, RI, USA.

**Table 2:** Characteristics of the participating women with OH-PCB measurements from the districts of Michalovce (*n* = 131) and Svidnik/Stropkov (*n* = 31).

	Michalovce (%)	Svidnik/Stropkov (%)
Age (years)		
< 21	17 (13.0)	7 (22.6)
21–29	88 (67.2)	17 (54.8)
≥ 30	24 (18.3)	7 (22.6)
Missing	2 (1.53)	0
Previous children (*n*)		
0	57 (43.5)	12 (38.7)
1	35 (26.7)	8 (25.8)
≥ 2	37 (28.2)	11 (35.5)
Missing	2 (1.53)	0
Body mass index (kg/m^2^)		
< 23	82 (62.6)	17 (54.8)
23–28	24 (18.3)	9 (29.0)
> 28	11 (8.40)	2 (6.45)
Missing	14 (10.7)	3 (9.68)
Smoking		
No	82 (62.6)	19 (61.3)
Yes	47 (35.9)	12 (38.7)
Missing	2 (1.53)	0
Education		
University	6 (4.58)	3 (9.68)
High school graduate	60 (45.8)	16 (51.6)
Lower education	63 (48.1)	12 (38.7)
Missing	2 (1.53)	0
Marital status		
Married	116 (88.6)	29 (93.6)
Single, widowed, or divorced	9 (5.34)	2 (6.45)
Missing	6 (4.58)	0

**Table 3:** Distributions of PCB congeners suggested to be precursors for OH-PCB metabolites, measured in serum from Slovak mothers sampled at time of delivery.

	5th percentile	25th percentile	Median	75th percentile	95th percentile	Mean ± SD
Michalovce (*n* = 131)						
Lipid (mg/mL)	7.80	9.44	10.2	11.3	13.8	10.5 ± 1.86
PCBs (ng/mL ww)						
CB-118	0.02	0.06	0.13	0.20	0.56	0.19 ± 0.24
CB-153	0.57	1.21	1.66	2.65	5.72	2.29 ± 1.98
CB-105	< 0.007	0.01	0.02	0.04	0.10	0.04 ± 0.06
CB-138	0.38	0.78	1.05	1.74	3.81	1.49 ± 1.38
CB-180	0.46	1.12	1.63	2.46	5.32	2.16 ± 1.88
CB-170	0.22	0.48	0.68	1.06	2.26	0.93 ± 0.81
∑PCBs	1.81	4.12	5.73	8.96	19.05	7.71 ± 6.60
Svidnik/Stropkov (*n* = 31)						
Lipid (mg/mL)	7.39	8.52	10.1	10.8	13.5	9.82 ± 2.27
PCBs (ng/mL ww)						
CB-118	0.02	0.05	0.07	0.13	0.22	0.09 ± 0.08
CB-153	0.44	0.65	0.92	1.24	1.94	1.05 ± 0.62
CB-105	< 0.007	0.01	0.01	0.02	0.04	0.02 ± 0.03
CB-138	0.27	0.39	0.55	0.86	1.32	0.67 ± 0.43
CB-180	0.32	0.57	0.93	1.17	1.71	0.92 ± 0.54
CB-170	0.16	0.25	0.40	0.47	0.77	0.40 ± 0.25
∑PCBs	1.54	2.29	2.82	4.32	6.34	3.45 ± 1.97

These distributions represent the full cohort of 1,103 mothers and were obtained using weights inversely proportional to the sampling probabilities. ∑PCBs includes CBs 28, 52, 101, 123/149, 118, 114, 153, 105, 138, 167, 156/171, 157, 180, 170, and 189.

**Table 4:** Distributions of OH-PCB metabolites measured in serum from Slovak mothers sampled at the time of delivery.

	5th percentile	25th percentile	Median	75th percentile	95th percentile	Mean ± SD
Michalovce (*n* = 131)						
Lipid (mg/mL)	7.80	9.44	10.2	11.3	13.8	10.5 ± 1.86
HPCs (ng/g ww)						
4-OH-CB107	0.01	0.02	0.03	0.05	0.15	0.05 ± 0.07
3-OH-CB153	0.02	0.05	0.07	0.11	0.27	0.10 ± 0.09
4-OH-CB146	0.03	0.06	0.11	0.18	0.47	0.17 ± 0.23
3'-OH-CB138	0.02	0.04	0.07	0.10	0.22	0.08 ± 0.08
4'-OH-CB130	< 0.003	< 0.003	0.003	0.01	0.01	0.01 ± 0.03
4-OH-CB187	0.07	0.11	0.20	0.36	0.85	0.31 ± 0.36
3'-OH-CB180	0.01	0.01	0.02	0.03	0.08	0.03 ± 0.03
4'-OH-CB172	0.01	0.02	0.04	0.07	0.15	0.06 ± 0.07
4-OH-CB193	0.01	0.01	0.02	0.03	0.08	0.03 ± 0.04
PCP^a^	0.24	0.41	0.66	1.04	3.24	1.02 ± 1.35
Sum of major OH-PCBs^b^	0.18	0.34	0.52	0.88	2.11	0.77 ± 0.85
∑OH-PCBs	0.20	0.36	0.55	0.95	2.28	0.83 ± 0.91
OH-PCBs:PCBs	0.05	0.07	0.09	0.12	0.18	0.10 ± 0.05
Svidnik/Stropkov (*n* = 31)						
Lipid (mg/mL)	7.39	8.52	10.1	10.8	13.5	9.86 ± 2.25
HPCs (ng/g ww)						
4-OH-CB107	0.004	0.01	0.02	0.03	0.05	0.02 ± 0.01
3-OH-CB153	0.01	0.02	0.04	0.05	0.12	0.05 ± 0.04
4-OH-CB146	0.02	0.04	0.05	0.07	0.16	0.06 ± 0.05
3'-OH-CB138	0.01	0.02	0.03	0.05	0.13	0.04 ± 0.04
4'-OH-CB130	< 0.003	< 0.003	< 0.003	0.004	0.01	0.00 ± 0.00
4-OH-CB187	0.04	0.07	0.11	0.13	0.32	0.12 ± 0.08
3'-OH-CB180	< 0.003	0.01	0.01	0.01	0.03	0.01 ± 0.01
4'-OH-CB172	0.01	0.01	0.02	0.03	0.06	0.03 ± 0.02
4-OH-CB193	0.004	0.01	0.01	0.01	0.03	0.01 ± 0.01
PCP^a^	0.24	0.37	0.54	0.72	1.98	0.74 ± 0.78
Sum of major OH-PCBs^b^	0.11	0.19	0.30	0.38	0.93	0.32 ± 0.22
∑OH-PCBs	0.12	0.21	0.32	0.43	1.01	0.35 ± 0.24
OH-PCBs:PCBs	0.04	0.07	0.08	0.11	0.16	0.10 ± 0.05

These distributions represent the full cohort of 1,103 mothers and were obtained using weights inversely proportional to the sampling probabilities.

a PCP was reported as measured.

b Major OH-PCBs were 4-OH-CB107, 3-OH-CB153, 4-OH-CB146, 3′ -OH-CB138, 4-OH-CB187, and 4′ -OH-CB172.
